# Visual memory in musicians and non-musicians

**DOI:** 10.3389/fnhum.2014.00424

**Published:** 2014-06-26

**Authors:** Ana Carolina Rodrigues, Maurício Loureiro, Paulo Caramelli

**Affiliations:** ^1^Dean of Undergraduate Studies, Federal University of Minas GeraisBelo Horizonte, Brazil; ^2^Department of Instruments and Singing, School of Music, Federal University of Minas GeraisBelo Horizonte, Brazil; ^3^Behavioral and Cognitive Neurology Research Group, Department of Internal Medicine, Faculty of Medicine, Federal University of Minas GeraisBelo Horizonte, Brazil

**Keywords:** visual memory, visual attention, neuroplasticity, cognition, musical training

## Abstract

Many investigations have reported structural, functional, and cognitive changes in the brains of musicians, which occur as a result of many years of musical practice. We aimed to investigate if intensive, long-term musical practice is associated with improved visual memory ability. Musicians and non-musicians, who were comparable in age, gender, and education, were submitted to a visual memory test. The test consisted of the presentation of four sets of stimuli, each one containing eight figures to be memorized. Each set was followed by individual figures and the subject was required to indicate if each figure was or was not present in the memorized set, by pressing the corresponding keys. We divided the test in two parts, in which the stimuli had greater or reduced semantic coding. Overall, musicians showed better performance on reaction times, but not on accuracy. An additional analysis revealed no significant interaction between group and any part of the test in the prediction of the outcomes. When simple reaction time was included as covariate, no significant difference between groups was found on reaction times. In the group of musicians, we found some significant correlations between variables related to musical practice and performance in the visual memory test. In summary, our data provide no evidence of enhanced visual memory ability in musicians, since there was no difference in accuracy between groups. Our results suggest that performance of musicians in the visual memory test may be associated with better sensorimotor integration, since although they have presented shorter reaction times, such effect disappeared when taken in consideration the simple reaction time test. However, given existing evidence of associations between simple reaction time and cognitive function, their performance in the visual memory test could also be related to enhanced visual attention ability, as has been suggested by previous studies, but this hypothesis deserves more investigation.

## INTRODUCTION

Recognition of the influence of music on cerebral function has incited neuroscientists and musicians to investigate the connections between these two areas since the 1990s. According to Münte et al. (2002), musicians represent an ideal model to investigate plastic changes in the human brain, considering the complexity of the stimulus – music – normally related to very high levels of exposure during musical practice.

Many investigations have reported structural and functional changes in the brains of musicians, involving several regions, such as auditory (Pantev et al., 1998), motor (Amunts et al., 1997), and somatosensory areas (Elbert et al., 1995), as well as brainstem (Musacchia et al., 2007) and hippocampus (Herdener et al., 2010), which occur as a result of many years of musical practice. Jäncke (2009), in a brief review, presents several results of interesting studies demonstrating brain plasticity in musicians.

The structural and functional neuroplastic processes demonstrated in the brains of musicians may influence their cognitive functioning, revealing differences in comparison to non-musicians. Several works (e.g., Standley and Hughes, 1997; Costa-Giomi, 1999; Hetland, 2000; Rauscher and Zupan, 2000; Vaughn, 2000; Anvari et al., 2002; Ho et al., 2003; Schellenberg, 2004, 2006; Gromko, 2005; Forgeard et al., 2008; Piro and Ortiz, 2009) have demonstrated associations between formal musical training in children and improvements in non-musical cognitive abilities, such as literacy, mathematics and visual–spatial reasoning, as well as general intelligence.

For instance, Hetland (2000), in a meta-analysis, showed that music instruction in childhood enhances performance on certain spatial tasks. Vaughn (2000), also in a meta-analysis, found a modest positive association between the study of music by children and mathematical achievement. Ho et al. (2003) evaluated visual and verbal memory abilities in children and found that those with musical training demonstrated better verbal but not visual memory than did their counterparts without such training. When the children were followed up after a year, those who had begun or continued musical training had significant verbal memory improvement. Children who discontinued the training did not show any improvement. In a longitudinal study, Schellenberg (2004) compared two groups of children who took music lessons (keyboard or voice) with two control groups who received drama lessons or no lessons. The study demonstrated that the first groups, in comparison with the controls, exhibited greater increases in full-scale IQ from pre-lesson to post-lesson periods, although the effect was relatively small. In correlational studies, Schellenberg (2006) found positive associations between the duration of music lessons in childhood and IQ among children aged 6–11 years, and similar but weaker correlations among undergraduates.

However, fewer studies have addressed the effects of musical practice on cognition in adults. Some of these investigations have demonstrated enhanced verbal memory ability in individuals with musical training (e.g., Chan et al., 1998; Kilgour et al., 2000; Brandler and Rammsayer, 2003; Franklin et al., 2008; Jakobson et al., 2008; Huang et al., 2010). Indeed, as pointed out by Jakobson et al. (2008), in many respects the skills underlying the ability to learn a piece of music resemble those involved in memorizing a poem or a piece of prose. In both cases, for instance, it is important not just to remember individual units of information (notes or words), but to recall them in the correct sequence.

Other investigations in adults have suggested the presence of enhanced visual cognition in adult musicians. Brochard et al. (2004) investigated visual–spatial abilities, using a neuropsychological test in which subjects were required to detect the position of a target dot relative to vertical and horizontal reference lines flashed on a screen. In one condition (perception condition), the reference line remained on the screen until the dot was displayed, while in a second condition (imagery condition), the line disappeared before the target dot was presented, requiring subjects to keep a mental image of the reference line. In both conditions, musicians exhibited shorter reaction times when compared to controls, suggesting the presence of augmented visual–spatial abilities in the former group. The comparison of saccadic eye movements in musicians and non-musicians also revealed important aspects of music reading practice. As noted by Kopiez and Galley (2002), the pattern of saccadic eye movements can be used as an indicator of mental disabilities, as well as a measure of mental processing speed. Kopiez and Galley (2002) and Gruhn et al. (2006) investigated saccadic eye movements during oculomotor tasks in adult subjects and reported more efficient oculomotor strategies in musicians when compared to non-musicians.

Patston et al. (2006) compared right-handed musicians and non-musicians in a line-bisection task. In this task, neurologically intact right-handers show a slight yet reliable tendency to bisect approximately 2% to the left of the true center, which has been attributed to the dominance of the right hemisphere for visual–spatial attention. These authors found that musicians showed a slight rightward bias, while non-musicians showed greater deviation to the left, and that musicians bisected the lines more accurately and with smaller intermanual differences than the control group. The researchers suggested that the left hemispheres of musicians may present an increased ability to perform cognitive functions that are usually right-hemisphere dominant, resulting in a more balanced visual–spatial attention. In another study, Patston et al. (2007) investigated the lateralization of visual attention in musicians and non-musicians, comparing reaction times and accuracy to stimuli presented to the left and right of a vertical line – a similar task to that used by Brochard et al. (2004). While both musicians and non-musicians performed more accurately for the left sided-stimuli, musicians were significantly more accurate than controls for the right-sided stimuli, and they also had faster reaction times overall. According to the authors, these results indicate a more balanced attentional capacity in musicians, as well as enhanced visual–motor ability, which is consistent with previous research.

Stoesz et al. (2007) demonstrated the presence of increased visual processing of local details in musicians when compared to non-musicians by utilizing disembedding and constructional tasks. According to the authors, this phenomenon may be related to changes in the neural system involved in controlling exploratory eye movements and shifts of visual attention. Jakobson et al. (2008) found a superior visual memory in musicians relative to non-musicians and hypothesized that this result could be due to improvements in supporting processes of visual attention, to the increased ability to hold and manipulate visual images in working memory, or even to the superior use of high-level strategic memory processes by musicians. In a previous study (Rodrigues et al., 2013), we evaluated three forms of visual attention ability – selective, divided, and sustained attention – in orchestra musicians and non-musicians by utilizing different neuropsychological tests, which measured accuracy and reaction times. Musicians showed better performance, when compared to non-musicians, on some variables of the three visual attention tests, suggesting that long-term musical training may be associated with enhancement in different forms of visual attention ability.

Moreover, studies involving brain imaging techniques have corroborated the behavioral evidences, suggesting more efficient visual processes in musicians (e.g., Sluming et al., 2002; Gaser and Schlaug, 2003; Schmithorst and Holland, 2003; Sluming et al., 2007; Groussard et al., 2010; Huang et al., 2010).

The aim of our study was to investigate whether intensive, long-term musical practice could be associated with improved visual memory abilities. As previously mentioned, some studies have investigated associations between musical training and visual cognition, but research specifically involving visual memory abilities is still scarce and controversial. While an investigation has suggested enhanced visual memory in musicians (Jakobson et al., 2008), other studies (Chan et al., 1998; Brandler and Rammsayer, 2003; Ho et al., 2003; Cohen et al., 2011) have not corroborated this hypothesis. Considering that musical practice involves such cognitive function as, in their professional routine, along with requirements of auditory memorization, musicians deal with the need of visual memorization of musical excerpts with different complexity levels, when reading a score, more investigations are needed. We compared the performance of musicians with that of non-musicians in a visual memory task by utilizing a neuropsychological test, designed for this investigation, and we hypothesized that musical training may be associated with improved mnemonic skills.

## MATERIALS AND METHODS

### PARTICIPANTS

Two groups of volunteers participated in the study: 38 musicians (mean age = 33.3 ± 7.6 years; 31 males and 7 females) and 38 non-musicians (mean age = 31.3 ± 5.6 years; 25 males and 13 females). The groups were comparable in terms of age [*t*(74) = 1.29; *p* = 0.200], gender [*X*^2^(1) = 2.44; *p* = 0.118] and education [*t*(74) = –0.59; *p* = 0.556], which was measured in terms of years of schooling, considered from basic education to post-graduation. The group of musicians consisted of 23 string players and 15 wind players, permanent members of two major Brazilian symphony orchestras, the Philarmonic Orchestra of Minas Gerais and the Symphony Orchestra of Minas Gerais. Both orchestras maintain a minimum weekly rehearsal schedule of 15 h and an intense annual concert program. Twenty musicians reported also playing a secondary instrument.

The daily time dedicated to individual musical instrument practice varied from 1 to 8.5 h (mean = 3.2 ± 1.2). The age at the commencement of musical studies ranged from 4 to 20 years (mean = 9.6 ± 4.4). The total musical training time and symphony orchestra practice time varied from 11 to 37 years (mean = 23.0 ± 6.7) and 4 to 26 years (mean = 13.9 ± 6.0), respectively.

The group of non-musicians consisted of professionals from different disciplines, as well as graduate and undergraduate students from several fields. All non-musicians reported that they were not able to read music scores. However, seven participants mentioned having received formal music lessons, although not for more than 2 years (mean time period of musical education = 8.5 ± 4.8 months), and five participants reported currently playing an instrument, but without regularity. All volunteers provided written consent to participate in the study, which was approved by the local ethics committee.

### PROCEDURE

Before the administration of the tests, we applied a sociodemographic questionnaire, to characterize each individual, and a segment of the Mini International Neuropsychiatric Interview (Sheehan et al., 1998), to investigate possible psychiatric disorders. None of the participants exhibited major depressive episode or alcohol dependence/abuse. Moreover, none of the subjects related using drugs with effects on central nervous system.

To evaluate the visual memory ability, we constructed a neuropsychological test using E-Prime software (Schneider et al., 2002). Visual stimuli were presented on a 1280 × 800-pixel computer screen, from which subjects were positioned at a distance of 55 cm. The participants accomplished the task with the preferred hand, and the answers were registered on the numeric keyboard of the computer.

**FIGURE 1:**
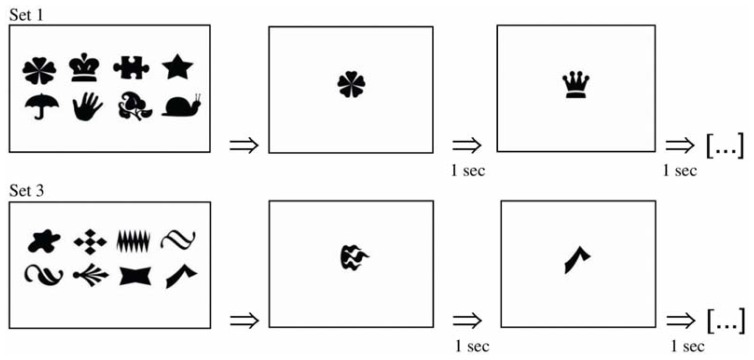
**Visual memory test’s schema.** Four sets of stimuli (only two are presented here) were exhibited on the screen during 10 s and were followed by 24 figures, which were individually and randomly exhibited on the center of the screen at intervals of 1 s. The subject had to respond if each figure was or was not present in the memorized set, by pressing the “1” or “2” keys, respectively.

The visual memory test (**Figure 1**) consisted of the presentation of four sets of stimuli, each one containing eight figures regularly arranged in two rows on the center of the screen. Each set was separately presented on the screen during 10 s and was followed by 24 figures, which were individually and randomly exhibited on the center of the screen. There was an interval of 1 s between the figures, which were (8 figures) or were not (16 figures) present in the previously displayed set. We opted for the exhibition of the figures presented in the set among 16 figures that were not present, in order to increase the number of stimuli that were similar, but not identical, to those presented in the set, thus enhancing the complexity of the task. All the figures were 3.0 cm high and 3.0 cm wide. In order to investigate the performance of the subjects in different levels of task difficulty, we divided the test in two parts: part 1 (first and second sets), in which the stimuli had greater semantic coding (e.g., car, heart, star) and part 2 (third and fourth sets), in which the stimuli had reduced semantic coding (e.g., abstract figures). There was no interval between each part of the test. The subject was required to indicate as quickly as possible whether each figure was or was not present in the memorized set, by pressing the “1” or “2” keys, respectively. Each figure was presented on the screen for a maximum of 5 s. If no response was given during this time interval, the next stimulus was presented after a pattern interval of 1 s. The measured variables were reaction time, considering only correct responses, and accuracy, both in the entire test, which presented 96 trials, and in the parts 1 and 2, each one presenting 48 trials. The test lasted approximately 2.5 min.

Because faster responses on the visual memory test could be explained by enhanced sensorimotor skills, subjects were submitted to a simple reaction time test to measure general motor coordination. According to Brochard et al. (2004), shorter simple reaction times would mean faster motor coordination, whatever the stimuli presented to the subjects. In the test, the subject was asked to press the “1” key as quickly as possible in response to the presentation of the symbol “*” of the size of 0.7 cm × 0.7 cm, flashing on the center of the screen at varying time intervals, ranging from 500 to 2500 ms. Throughout the test, the symbol was presented 40 times during a maximum period of 3 s. If the subject did not respond during this interval, the next stimulus was presented after a varying period of time. The measured variable was reaction time. The test consisted of 60 trials and lasted approximately 1 min.

Each test was preceded by standardized instructions. Reaction time was measured in milliseconds and accuracy was measured in the percentage of correct responses.

### STATISTICAL ANALYSIS

After testing the normality of each variable, utilizing the Kolmogorov–Smirnov test, mean comparisons between musicians and non-musicians were performed using the Student’s *t*-test for independent samples. Effect sizes (Cohen’s *d*) were calculated for the comparisons that revealed significant differences between groups. A two-way mixed design ANOVA was run to investigate possible interactions between group (musicians and non-musicians) and part of the visual memory test (parts 1 and 2), with group entered as the between-subjects factor and test’s part entered as the within-subjects factor. ANCOVA was also performed between groups using simple reaction time as covariate. Correlations between the performance of musicians in this test and variables related to musical practice – age at the commencement of musical studies, daily individual instrumental practice and number of years of musical practice – were also calculated using Pearson’s correlation test, for data with normal distribution, and Spearman’s correlation test, for data without normal distribution. Gender was compared, between musicians and non-musicians, using the Chi-square test. The significance level was set to 5% (*p* < 0.05) for all tests.

## RESULTS

**FIGURE 2:**
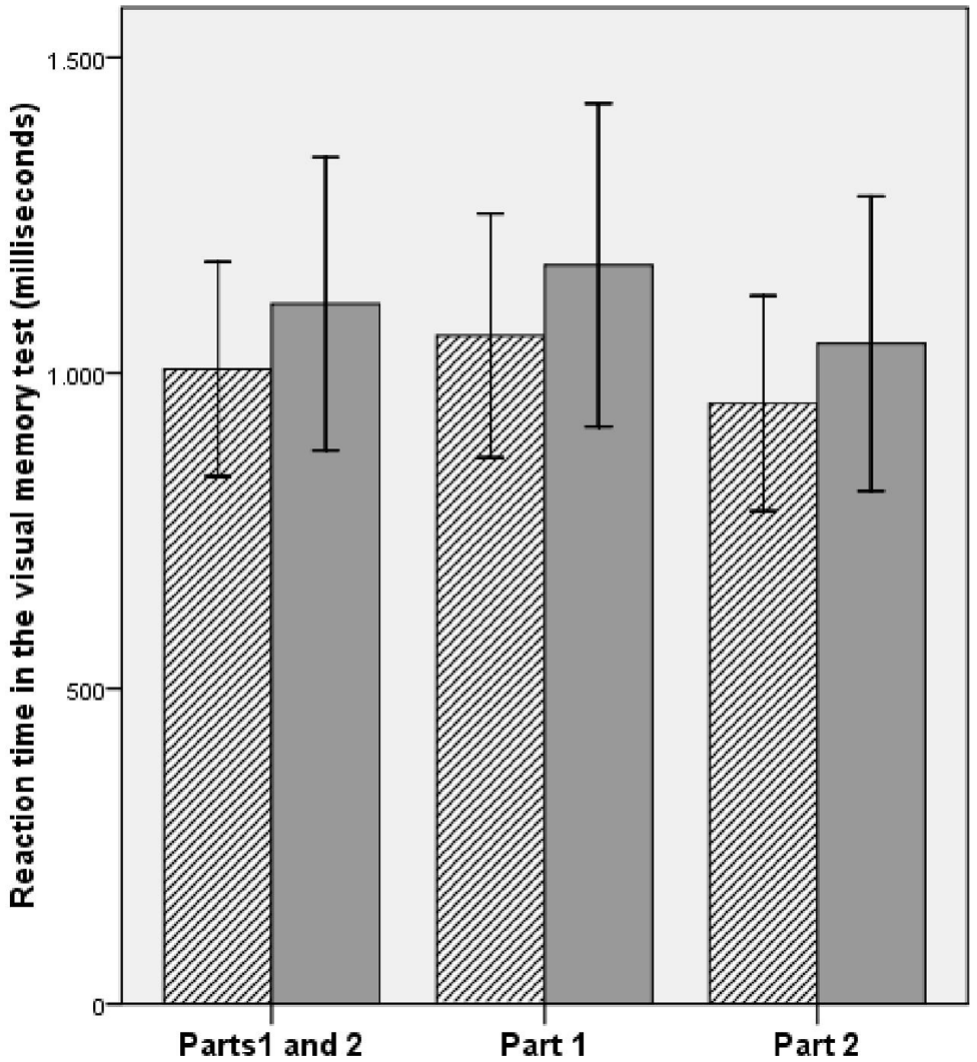
**Reaction time in the visual memory test (parts 1 and 2, part 1 and part 2) in musicians (crosshatched) and non-musicians (gray).** Musicians performed better than non-musicians in all three situations (*p* < 0.05). Error bars represent ±1 SD.

As shown in **Table 1**, musicians performed better in three variables of the visual memory test, namely, reaction time in the entire test [*t*(74) = –2.20; *p* = 0.030], reaction time in part 1 [*t*(74) = –2.15; *p* = 0.035], and reaction time in part 2 [*t*(74) = –2.01; *p* = 0.048; **Figure 2**]. The effect size (*d*) for each of the differences mentioned above was 0.50, 0.49, and 0.46, respectively. These values are between the effects considered small (0.20) and medium (0.50) by Cohen (1988). Accuracy was similar between groups in all tasks. No difference between musicians and non-musicians was observed in the simple reaction time test [*t*(74) = –1.86; *p* = 0.067], although there was a tendency toward shorter reaction times in the group of musicians.

**Table 1:** Comparison between musicians and non-musicians in the visual memory test and in the simple reaction time test.

Test	Variable	Musicians (*n* = 38)		Non-musicians (*n* = 38)		*p*	Effect size (*d*)
		Mean	SD	Mean	SD		
Visual memory (Parts 1 and 2)	Reaction time	1006	170	1109	232	**0.030**	0.50
	Accuracy	82.40	5.90	83.24	6.07	0.540	0.18
Visual memory (Part 1)	Reaction time	1059	193	1171	256	**0.035**	0.49
	Accuracy	83.33	7.92	84.37	7.60	0.561	0.14
Visual memory (Part 2)	Reaction time	952	170	1046	233	**0.048**	0.46
	Accuracy	81.46	6.69	82.12	6.15	0.657	0.16
Simple reaction time	Reaction time	342	51	375	95	0.067	0.43

*The p values refer to the Student’s t-test for independent samples. The black p values indicate significant differences at a 5% level. Reaction time is shown in milliseconds, and accuracy is shown in the percentage of correct answers.*

In order to investigate possible interactions between group (musicians and non-musicians) and part of the visual memory test (parts 1 and 2), we performed a two-way mixed design ANOVA, with group entered as the between-subjects factor and test’s part entered as the within-subjects factor. Considering reaction times, the analysis showed a main effect of group [*F*(1,74) = 4.90; *p* = 0.030], with shorter reaction times in the group of musicians, as demonstrated after conducting Student’s *t*-test (**Table 1**), a main effect of test’s part [*F*(1,74) = 47.90; *p* = 0.000], with shorter reaction times in part 2 for both musicians and non-musicians, and no interaction between group and test’s part [*F*(1,74) = 0.27; *p* = 0.601]. Considering accuracy, the analysis showed no main effect of group [*F*(1,74) = 0.38; *p* = 0.538], as demonstrated after conducting Student’s *t*-test (**Table 1**), a main effect of test’s part [*F*(1,74) = 5.38; *p* = 0.023], with higher percentage of correct responses in part 1 for both musicians and non-musicians, and no interaction between group and test’s part [*F*(1,74) = 0.04; *p* = 0.829].

Because there was a tendency to differences between the groups in the simple reaction time test, we performed an ANCOVA with this covariate, comparing performance of musicians and non-musicians in the visual memory test. We entered group (musicians and non-musicians) as the between-subjects factor, test’s part as the within-subjects factor and simple reaction time as the covariate. Considering reaction times, the analysis showed no main effect of group [*F*(1,73) = 2.02; *p* = 0.160], no main effect of test’s part [*F*(1,73) = 3.96; *p* = 0.050], and no interaction between group and test’s part [*F*(1,73) = 0.40; *p* = 0.527]. Considering accuracy, the analysis also showed no main effect of group [*F*(1,73) = 1.81; *p* = 0.182], no main effect of test’s part [*F*(1,73) = 2.17; *p* = 0.145], and no interaction between group and test’s part [*F*(1,73) = 0.18; *p* = 0.670].

**FIGURE 3:**
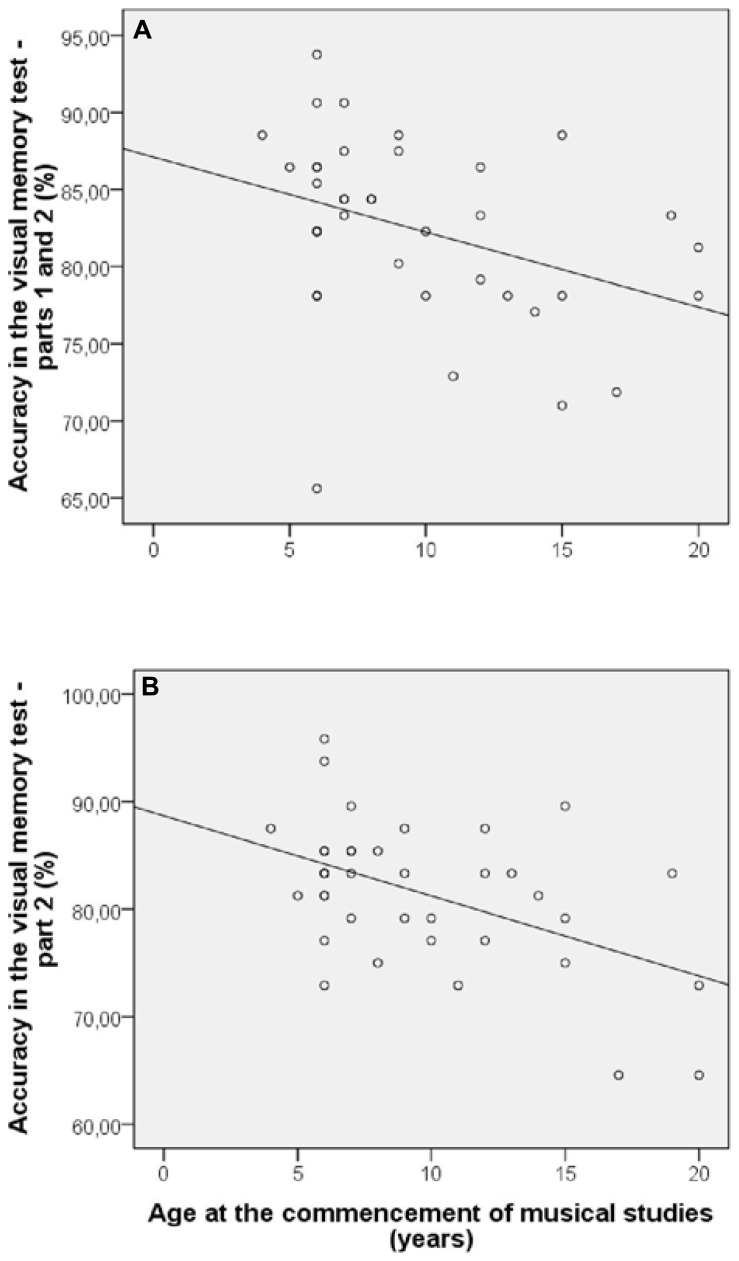
**Significant correlations (*p* < 0.05) between age at the commencement of musical studies and accuracy in the visual memory test – parts 1 and 2 (A) and part 2 (B)**.

**FIGURE 4:**
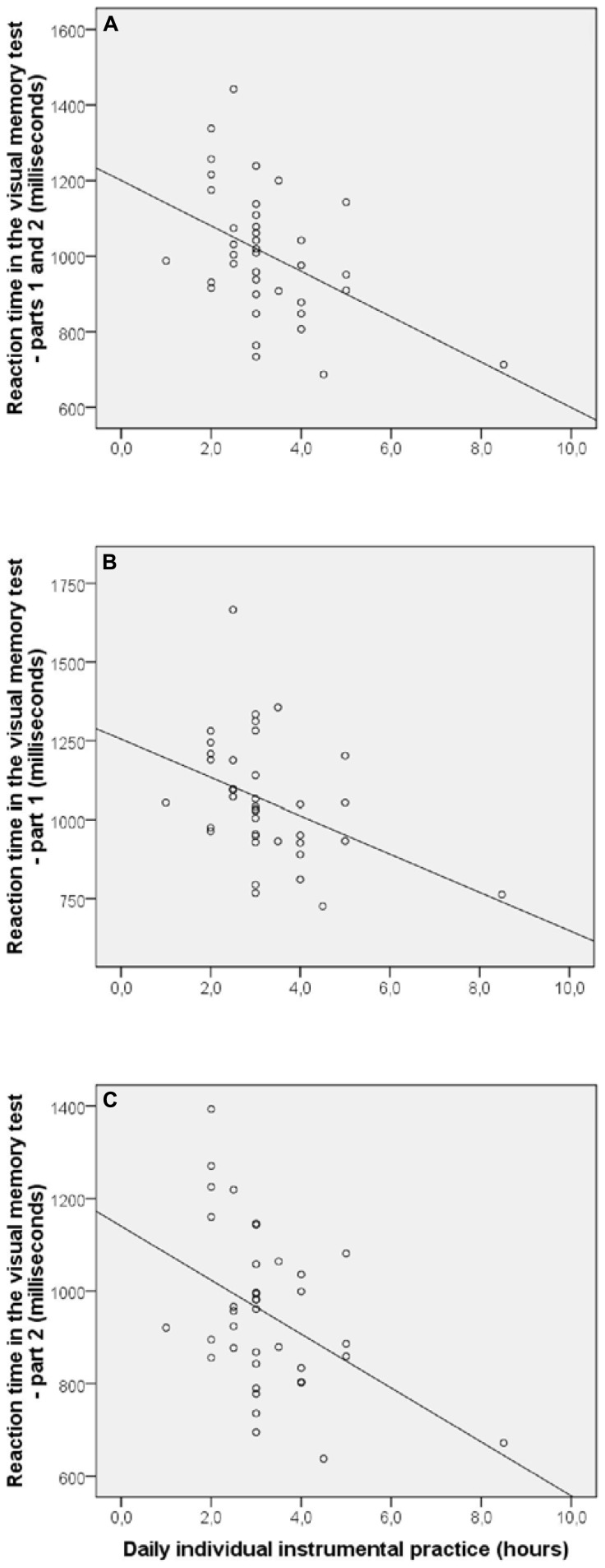
**Significant correlations (*p* < 0.05) between daily individual instrumental practice and reaction time in the visual memory test – parts 1 and 2 **(A)**, part 1 **(B)**, and part 2 (C)**.

In the group of musicians, significant correlations between the age at the commencement of musical studies and the performance in visual memory test were observed for two variables: accuracy in the entire test [*r*(36) = –0.36; *p* = 0.024] and accuracy in part 2 [*r*(36) = –0.49; *p* = 0.002; **Figure 3**]. Moreover, there were significant correlations between the daily individual instrumental practice and three variables: reaction time in the entire test [*r*(36) = –0.44; *p* = 0.005], reaction time in part 1 [*r*(36) = –0.46; *p* = 0.004], and reaction time in part 2 [*r*(36) = –0.37; *p* = 0.019; **Figure 4**]. Such correlations suggest a better performance in the visual memory test by musicians who began their musical studies at an early age and those who have a more intensive musical practice. There was no significant correlation between number of years of musical practice and performance of musicians in visual memory test. We also observed a significant correlation between the age when beginning musical studies and reaction time in the simple reaction time test [*r*(36) = 0.43; *p* = 0.007]. The results of all the correlation’s tests are presented in **Table 2**.

**Table 2:** Correlations between performance of musicians in the visual memory test and in the simple reaction time test and variables related to musical practice.

		Age at the commencement of musical studies		Daily individual instrumental practice (h)		Number of years of musical practice^*^	
Test	Variable	Correlation coefficient (*r*)	*p*	Correlation coefficient (*r*)	*p*	Correlation coefficient (*r*)	*p*
Visual memory (Parts 1 and 2)	Reaction time	0.15	0.352	-0.44	**0.005**	-0.15	0.369
	Accuracy	-0.36	**0.024**	-0.28	0.080	0.15	0.374
Visual memory (Part 1)	Reaction time	0.25	0.127	-0.46	**0.004**	-0.20	0.224
	Accuracy	-0.20	0.207	-0.24	0.147	-0.00	0.959
Visual memory (Part 2)	Reaction time	0.06	0.683	-0.37	**0.019**	-0.07	0.681
	Accuracy	-0.49	**0.002**	-0.22	0.177	0.28	0.083
Simple reaction time	Reaction time	0.43	**0.007**	0.08	0.608	-0.17	0.302

The correlations involving age at the commencement of musical studies and number of years of musical practice were measured by Pearson’s correlation test. The correlations involving daily individual instrumental practice were measured by Spearman’s correlation test. ^*^The correlation’s tests involving this variable were run controlling statistically for the variable “age,” since subjects with more years of musical practice are presumably older and therefore more susceptible to age-associated cognitive decline. The black p values indicate significant correlations at a 5% level.

## DISCUSSION

Musicians showed shorter reaction times, both in the entire visual memory test, and in each one of its parts: part 1 (stimuli with greater semantic coding) and part 2 (stimuli with reduced semantic coding), when compared to non-musicians. However, the effect sizes measured for each of the differences between musicians and non-musicians were not considered large, according to Cohen’s parameters (Cohen, 1988), ranging from small to medium. Moreover, the advantage of musicians relative to non-musicians could be at least partially explained by better sensorimotor integration, since there was a tendency toward shorter reaction times in the simple reaction time test in the group of musicians.

When investigating possible interactions between group (musicians and non-musicians) and part of the visual memory test (parts 1 and 2), after performing a two-way mixed design ANOVA, we first found a main effect of test’s part for both reaction times and accuracy. Musicians and non-musicians showed shorter reaction time in part 2, when compared to part 1, which can be associated with reduced semantic coding of the stimuli in the second part of the test. Although this fact increases the level of task difficulty, which was confirmed by the reduced accuracy of musicians and non-musicians in part 2 when compared to part 1, the decision of the subject may become faster, but not necessarily more accurate, due to the lower possibility of associations between stimuli. Similarly, to our results with Student’s *t*-test, the two-way mixed design ANOVA also showed a main group effect for reaction times, with musicians faster than non-musicians overall, and no main group effect for accuracy. Interestingly, the analysis revealed no interaction between group and test’s part for both reaction times and accuracy, which suggests that the increase of task difficulty level, from part 1 to part 2 in the visual memory test, had the same impact in performance of musicians and non-musicians.

Brochard et al. (2004) did not observe significant difference between musicians and non-musicians in a simple reaction time control test, which presented visual stimuli. However, as music score reading involves converting visual inputs into motor actions, shorter reaction times in the visual memory test could be attributed to better sensorimotor skills, which are developed during the intensive practice of a musical instrument. Although our investigation did not reveal significant difference between groups in the simple reaction time test, it is important to note that there was a tendency toward shorter reaction times in the group of musicians (*p* = 0.067), which does not allow us to disregard the role of faster sensorimotor integration in the improved performance in the visual memory test. It is noteworthy that the variability in the group of non-musicians was considerably larger than in the group of musicians, which may have accounted for the difference between groups. It is also important to mention that the larger variability in the group of non-musicians might be due to different levels of musical experience reported by the participants in this group. This issue can be further explored in future investigations.

Nevertheless, a tendency toward shorter reaction times in the simple reaction time test was an interesting observation. Several studies (e.g., Jakobsen et al., 2011; Cumming et al., 2012) have demonstrated associations between performance in simple reaction time tests and cognitive function. Jakobsen et al. (2011) investigated the validity of reaction time as a simple tool to measure cognitive function in healthy subjects and hospitalized patients, and showed that the simple reaction time test was correlated with some cognitive functions in both groups. Similarly, Cumming et al. (2012) investigated patients with acute stroke and demonstrated that performance in a simple reaction time test at baseline was associated with attentional function at 3 months post-stroke. Thus, taken together, the simple reaction time appears to be not only related to sensorimotor integration, but also to cognitive function in general. Therefore, the tendency toward shorter reaction times in the simple reaction time test may be expected in musicians, given the intensive sensorimotor training, as well as all the cognitive requirements, in their professional routine. The investigation of simple reaction time and its associations with other cognitive functions in musicians and non-musicians has great potential for future studies.

After performing ANCOVA for reaction times in the visual memory test, using simple reaction time as a covariate, we observed no differences between musicians and non-musicians. This result was in contrast to the results obtained after performing Student’s *t*-test, which indicated that the difference in the reaction times in the visual memory test disappeared when the simple reaction time was held constant, suggesting that the performance of the musicians in such a memory test may be related to their ability in the simple reaction time test. Consistent with previous studies (Jakobsen et al., 2011; Cumming et al., 2012), our results from ANCOVA favored a potential association between simple reaction time and cognitive function.

Considering that musical practice often requires memorizing a wide variety of visual symbols involved in music memorization as well as in music reading, it would be reasonable to expect a positive effect of musical training on visual memory abilities. Because the significant differences between groups only involved reaction times, which disappeared when performance in the simple reaction time test was held constant, it is possible to conceive that the better performance of musicians in the visual memory test reflects a better sensorimotor integration, as mentioned above. However, given the evidence of associations between simple reaction time and cognitive function (Jakobsen et al., 2011; Cumming et al., 2012), the performance of musicians in the visual memory test could also be related to greater efficiency of attentional processes, as has been suggested in previous studies (e.g., Rodrigues et al., 2013). It is possible to argue that music reading practice may contribute to enhanced visual attention ability in general. As indicated by Land and Furneaux (1997), this practice involves a large amount of processing of the input signal – pitch, duration, timing, and dynamics of notes have to be decoded – also requiring great competence in the execution of the synchronized motor output. According to these authors, music reading is notably a more structured process when compared to text reading. In relation to eye movements, music reading involves longer fixations, with less regular durations than in text reading. In general, fixations are longer when music presents a higher level of melodic or rhythmic difficulty. Thus, a new saccadic eye movement would occur only after information arising from a previous fixation had been processed (Kinsler and Carpenter, 1995). Therefore, the practice of music reading involves cognitive aspects, including the considerable attention needed for visual stimuli processing. Thus, although musicians and non-musicians have performed the visual recognition task with similar accuracy, maybe musicians were more attentive to the stimuli, focusing the attention on the characteristics of each visual stimulus, selecting the action to correctly respond each trial and maintaining the alertness state, thus producing faster responses. However, more specific tests are necessary to investigate this hypothesis.

Seemingly, our results observed in accuracy are not consistent with those of Jakobson et al. (2008), which suggested enhanced visual memory abilities in musicians, demonstrated in recall and recognition tasks. However, it is necessary to point out that the nature of the test used by those researchers differs from that of our study. While in the study of Jakobson et al. (2008) the stimuli to be memorized were simple geometric figures, which were sequentially presented, in our investigation the stimuli were more complex figures, which had different levels of semantic coding and were displayed in sets of eight different figures. Moreover, while in the test of Jakobson et al. (2008) the recognition task occurred 15 min after the presentation of the figures, in our test such task was performed immediately after the exhibition of the stimuli set. Thus, maybe musical training has a greater effect on the memorization ability of certain kinds of visual stimuli, and/or when such ability involves a longer retention time. Moreover, in the study of Jakobson et al. (2008), although the participants were not professional musicians, as they are in our study, they initiated their music lessons at a mean age of 5.8 years (SD = 1.4; range: 3–9 years), an early age when compared to our study, in which the musicians started at a mean age of 9.6 years (SD = 4.4; range: 4–20 years). Thus, this fact may have contributed to the difference between the results.

On the other hand, although Jakobson et al. (2008) have found positive results, other studies (e.g., Chan et al., 1998; Brandler and Rammsayer, 2003; Ho et al., 2003; Cohen et al., 2011) did not observe effects of musical training on visual memory. However, some aspects of these studies may have contributed to this apparent discrepancy. As also pointed by Jakobson et al. (2008), Chan et al. (1998), and Ho et al. (2003) investigated Asian samples, that are traditionally trained in the use of an ideographic writing system, which may be associated with better memory for abstract designs (Flaherty, 2000). Thus, such kind of training may have attenuated possible benefits of musical training on visual memory ability. Brandler and Rammsayer (2003), although have not worked with Asian samples, utilized a visual memory test that required topographical abilities, which may involve different neural substrates, compared to those supporting visual memory for faces and designs (e.g., Mecklinger, 1998).

Cohen et al. (2011) used a test in which subjects had to memorize, in the study phase, several sequentially presented visual stimuli – objects and abstract art pieces. During the test phase, participants were presented with another set of stimuli, of which half were images they had seen in the study phase, and they had to classify each stimulus as “old” or “new.” It is important to stress that this test, as the one used in our study and different from that one applied by Jakobson et al. (2008), involved visual stimuli of a greater complexity, which may have contributed to the divergence between the results. It is also necessary to point out that, as in our investigation, the visual memory test exhibited stimuli with greater (objects) or reduced semantic coding (abstract art pieces) and, similarly to our results, the authors did not find significant differences between musicians and non-musicians in the recognition of each kind of stimulus. Moreover, as in our study and in opposition to the test used by Jakobson et al. (2008), the recognition task was performed immediately after the presentation of the stimuli.

Therefore, data about effect of musical training on visual memory ability are not consistent yet, thus requiring more investigation. However, it is important to stress that some studies involving brain imaging techniques may suggest a greater efficiency of mnemonic processes in musicians (e.g., Gaser and Schlaug, 2003; Groussard et al., 2010; Herdener et al., 2010; Huang et al., 2010).

Regarding the significant correlations found between variables related to musical practice and performance in the visual memory test, the results suggest that musicians who began their musical studies at an early age and who have a more intensive musical practice tend to have more efficient mnemonic and attentional processes, respectively. We could suggest these associations since the correlations involving age at commencement of musical studies were observed on accuracy, while those involving daily individual instrumental practice were found on reaction times. Thus, higher percentage of correct responses would reflect a greater ability to memorize the stimuli’s set, and shorter reaction times would demonstrate enhanced attention ability. This increased cognitive efficiency may be related to processes of cerebral neuroplasticity. Several works have demonstrated significant correlations between the age of initiation of musical studies (e.g., Elbert et al., 1995; Schlaug et al., 1995; Amunts et al., 1997; Pantev et al., 1998) and the intensity of musical practice (e.g., Gaser and Schlaug, 2003; Bengtsson et al., 2005) and cerebral neuroplastic processes. The correlations that we observed could suggest the existence of an adaptive process as a result of increased long-term sensorial stimulation. However, to establish more objective associations further evidence is needed, as the correlations were observed for only some variables. There was no significant correlation between number of years of musical practice and performance of musicians in visual memory test. We could suggest that maybe the age of beginning of musical studies as well as the intensity of musical practice, rather than its duration, have a greater effect on cognition. However, since there is evidence of significant correlations between number of years of musical practice and degree of structural and functional changes in the brain (Sluming et al., 2002; Musacchia et al., 2007; George and Coch, 2011), our results cannot be considered conclusive.

Our study has several limitations. First, we did not control for general intelligence (IQ), as previous studies addressing possible effects of musical training on cognitive abilities have controlled for (e.g., Franklin et al., 2008; Bialystok and DePape, 2009; Schellenberg and Moreno, 2009). However, these studies have not found differences in IQ between musicians and non-musicians. Second, our group of non-musicians included some individuals with musical experience. However, other authors (e.g., Franklin et al., 2008; Jakobson et al., 2008; Strait et al., 2010; George and Coch, 2011) have also considered these subjects in their analysis. It is important to note that, in our study, the musical experience was limited to a short time period of musical education (mean = 8.5 ± 4.8 months), occurred in the past, or to an occasional instrumental practice, criteria that are similar to those used in previous studies. More importantly, considering that our aim was to investigate a visual cognitive ability, none of the non-musicians were able to read music scores. Third, we did not apply standardized neuropsychological tests to investigate the visual memory ability. Rather, we constructed a test seeking to present a wide variety of visual stimuli, as well as to enable accurate recording of reaction times, often unmeasured variable in conventional assessment instruments. Although other studies have also applied non standardized tests to compare visual cognitive abilities in musicians and non-musicians (e.g., Brochard et al., 2004; Patston et al., 2007), we must recognize the relevance of utilizing tests already validated for the population in general.

Fourth, the visual memory test and the simple reaction time test were not comparable, in terms of stimuli presentation. One could argue that differences between the performance of subjects in these two tests might be due to differences in stimuli presentation, rather than to cognitive components evaluated by each test. However, in the study of Brochard et al. (2004), the tests were comparable and the results were similar to those observed in our investigation. Finally, as indicated by Schellenberg and Peretz (2007), the question of causation is an important issue related to music and cognition that remains unresolved. It is not yet known whether augmented cognitive abilities, demonstrated in musicians, are in fact a consequence of long-term training, or whether they are inborn. Because most of the studies that suggest benefits of musical practice on cognition, including this investigation, are of a correlational or quasi-experimental nature, an objective establishment of a clear causal link is not possible. Therefore, more experimental designs are required to determine causation.

In summary, our data provide no evidence of enhanced visual memory ability in musicians, since there was no difference in accuracy between groups. Our results suggest that performance of musicians in the visual memory test, when compared to non-musicians, may be associated with better sensorimotor integration, since although musicians have presented shorter reaction times, such effect disappeared when taken in consideration performance in the simple reaction time test. Nevertheless, given existing evidence of associations between simple reaction time and cognitive function, the performance of musicians in the visual memory test could also be related to greater efficiency of attentional processes, as has been suggested by previous studies. However, this hypothesis deserves more investigation. This study may stimulate further research addressing the effects of musical training on visual memory abilities, a still controversial issue in the literature devoted to the study of the influence of music on brain and cognition.

## Conflict of Interest Statement

The authors declare that the research was conducted in the absence of any commercial or financial relationships that could be construed as a potential conflict of interest.
